# Dentistry and dental care in antiquity: part 1 – prehistory, Mesopotamia, Israel, Etruria and the Far East

**DOI:** 10.1038/s41415-025-8884-z

**Published:** 2025-12-19

**Authors:** Roger Forshaw

**Affiliations:** KNH Centre for Biomedical Egyptology, Faculty of Biology, Medicine and Health, Oxford Road, The University of Manchester, Manchester, M13 9PL, United Kingdom

## Abstract

This paper – the first of two – explores the development of dentistry and dental care practices across diverse ancient civilisations. Evidence from prehistory, from a 13,000-year-old intervention at Riparo Fredian in northern Italy, to Neolithic findings in Pakistan and Slovenia, suggests that early populations attempted to alleviate pain and manage oral conditions. In Mesopotamia, cuneiform texts detail treatments for caries and periodontal disease, accompanied by recommendations and prescriptions for oral hygiene. Although these texts describe various therapeutic approaches, there is no mention of any operative procedures, and the sparse osteological record similarly offers no evidence of dental intervention. Biblical and Talmudic sources from ancient Israel emphasise the cultural significance of dental aesthetics, offering insights into remedies and practices intended to preserve the natural look of the teeth. Discoveries from Etruria and Phoenicia, dated to the first millennium BC, including dental bridges and gold-wire appliances, reveal intricate restorative and cosmetic techniques, particularly among elite women. In the Far East, ancient Chinese and Indian texts highlight preventive measures and herbal treatments, prioritising diagnostics and hygiene over operative procedures. Collectively, these findings illustrate a broad spectrum of early dental care strategies that evolved, alongside dietary shifts, cultural values, and technological innovations, providing fascinating insights into the origins and development of dentistry and dental care.

## Key points

- Gain an overview of how early humans transitioned from rudimentary dental interventions in prehistory to more sophisticated treatment approaches in later ancient civilisations.
- Explores the multifaceted dental practices across Mesopotamia, Israel, the Mediterranean and the Far East, highlighting how empirical remedies intertwined with ritualistic elements.
- Illustrates how cultural practices and dietary changes influenced oral health, providing insights into the evolutionary roots of modern dental care.

## Prehistoric dental intervention

During prehistory, the transition from a hunter-gatherer lifestyle to a mixed agricultural economy marked a pivotal change in human dietary practices, notably characterised by a significant increase in carbohydrate consumption. This dietary shift coincided with a rise in dental caries and other oral pathologies.^1^ Evidence of rudimentary prehistoric dentistry aimed at addressing these conditions is sparse yet intriguing, and although these cases offer insights into early therapeutic interventions, alternative explanations for the evidence cannot be discounted.

The earliest known case of possible operative dental work, dating to approximately 14,000 years ago, was discovered in a young male from the archaeological site of Riparo Villabruna in northern Italy.^2^ The right mandibular third molar exhibits a large, intentionally prepared occlusal cavity with some residual carious tissue. Detailed analyses, including scanning electron microscopy, revealed striations on the internal walls extending into the base of the cavity, which had morphological similarities to cutmarks on bone.^3^ Experimental tests indicated that these striations were likely produced by microlithic flint points, and could represent a primitive attempt at carious removal to alleviate dental pain.

Another case from Italy, dated to a similar period, was identified at the Palaeolithic site of Riparo Fredian. Analysis of two isolated upper incisors revealed significant wear and antemortem enlargement of their pulp chambers, as evidenced by striations on their inner walls. Scratches and rounded fractures along the cavity margins indicate that the teeth continued to function after the procedure. Moreover, scanning electron microscopy detected traces of bitumen and organic fibres within the chambers, possibly suggesting an attempt at a rudimentary filling material. Together, these findings lend support to the hypothesis that the procedure served as a therapeutic intervention aimed at alleviating pain from tooth sensitivity or pulpitis.^4^

In addition to these operative approaches, potential evidence of non-invasive dental hygiene practices has also been discovered. Interproximal grooves found at the cementoenamel junction on premolar and molar teeth have been documented from ancient times to the present.^5^^,^^6^^,^^7^ Although theories such as fibre or sinew processing have been proposed, the grooves' distinct shape and placement are more consistent with early humans using a primitive toothpick to remove trapped food particles. While it is likely that not all interproximal grooves are caused by probes, it would seem that in many cases the use of toothpicks would appear to be a reasonable explanation.^6^ This finding implies that prehistoric communities were removing trapped food particles not only for comfort but may also have been attentive to oral hygiene long before the emergence of modern dental care.

Further evidence comes from a Neolithic graveyard in Pakistan, dating from approximately 7,500–9,000 years ago, where researchers uncovered 11 molar teeth with intentionally prepared cavities. While four of these teeth displayed carious lesions, the remaining specimens showed no decay, and all lacked traces of any restorative filling material. Microscopic analyses revealed drill-like marks within the cavities. Flint drill heads, likely used for bead making, were also recovered from the graveyard, and experimental replication confirmed their ability to create similar cavities. The functional significance of this practice remains uncertain, particularly given that subsequent populations at the site did not continue this procedure.^8^

Lastly, on a canine from a 6,500-year-old mandible discovered at a Neolithic site in Slovenia, researchers identified traces of beeswax. The tooth, which exhibited significant wear and a vertical crack extending through both enamel and dentine, may have been treated with beeswax to relieve tooth sensitivity.^9^ However, it remains unclear whether the application occurred ante-mortem or resulted from an unconnected post-mortem event.

Collectively, these cases provide limited evidence of prehistoric dental intervention. They suggest that early humans may have employed rudimentary therapeutic techniques to address dental pathologies and engaged in preventive measures to maintain oral hygiene. Further research is necessary to clarify the intentionality and prevalence of these practices, ultimately enhancing our understanding of the evolution of early dentistry.

## Mesopotamia

**Fig. 1:**
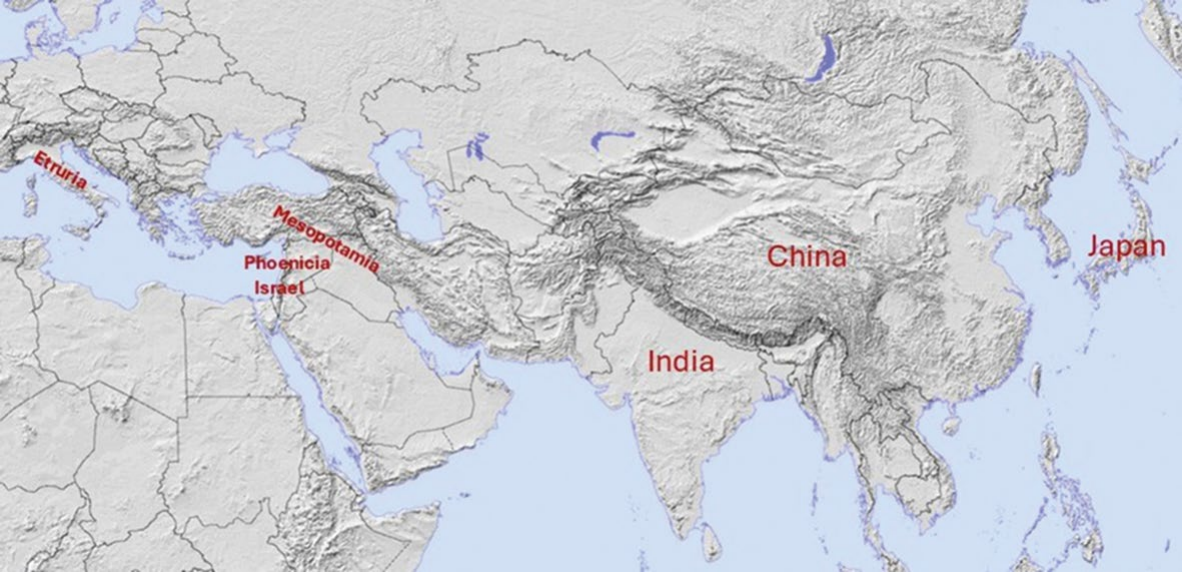
Map of the ancient civilisations. Copyright ©2010, 2013 by Ian Macky

Mesopotamian society emerged around 12,000 BC along the banks of the Tigris and Euphrates rivers, a region often hailed as the cradle of civilisation (Fig. 1). The Sumerians represent the earliest identifiable culture in this area, later succeeded by the Babylonians and Assyrians.

**Fig. 2:**
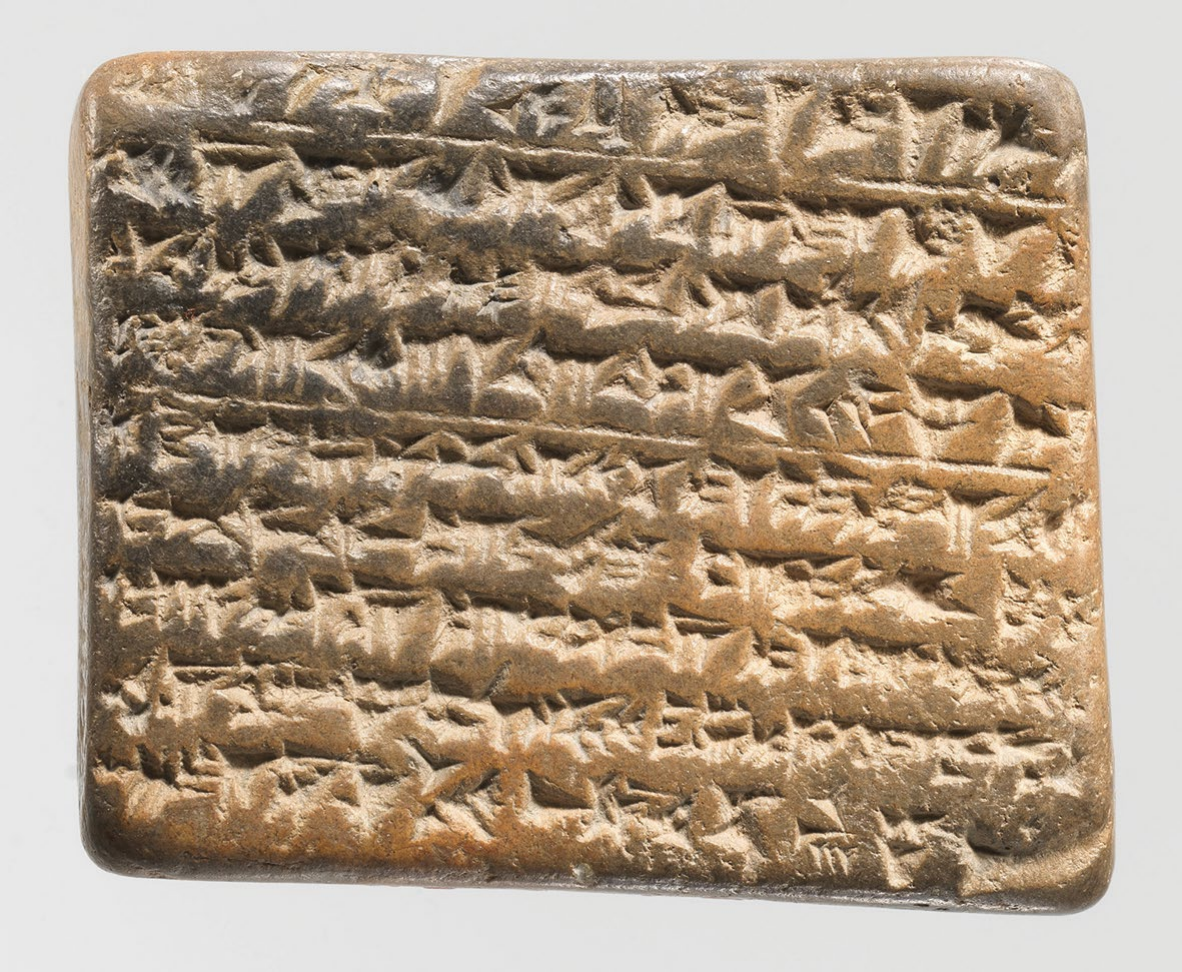
Clay cuneiform tablet (56.81.52): a fragment of a medical text from Assyria, dating to the 9^th^ to the 7^th^ century BC. Courtesy of the Metropolitan Museum of New York. Credit: Rogers Fund. Public Domain

A substantial collection of Sumerian, Babylonian and Assyrian texts has survived, preserved in cuneiform script on thousands of clay tablets. Many of these texts were compiled into a library by the Assyrian King Ashurbanipal around 700 BC, though many are believed to be copies of earlier works^10^ (Fig. 2). Among them are writings that explore both medical and dental topics.

Over 100 prescriptions, with many incorporating incantations, detail treatments for various oral conditions. The remedies primarily relied on botanical and mineral-based compounds, with only limited use of animal-derived products. The prominence of oral hygiene is underscored by numerous prescriptions aimed at alleviating bad breath and other oral ailments, with these frequently accompanied by magical incantations. Some texts describe the use of mouthwashes, while one records a method of tooth cleaning using an index finger wrapped in cloth soaked in a mixture of alum, salt, and vinegar, arguably the earliest recorded instance of dental hygiene.^11^

Other remedies for dental care included scraping teeth to remove stains and, remarkably, a formula for whitening discoloured teeth: ‘if a man's teeth have become yellow, his mouth…him…though shall grind salt of Akkad, amni, pine turpentine and thy finger thou shall rub his teeth…'.^12^

Archaeological evidence further highlights the significance of oral care. Toothpicks, some dating as far back as 3500 BC, suggest an early awareness of dental hygiene. This importance is reinforced by discoveries from the Ningal Temple in the ancient city of Ur, where elaborate cosmetic sets, which included silver or gold tweezers, ear scoops, and toothpicks, have been recovered. Moreover, their presence as grave goods intended for use in the afterlife indicates their cultural and practical value.^13^

Caries was often attributed to a ‘tooth worm', leading to treatments that combined therapeutic compounds with incantatory rituals. For example, one remedy involved heating a mixture of henbane seed and beeswax and directing the resulting smoke into the affected cavity. Periodontal disease was also recognised, with treatments involving gum massages using various compounds, perhaps the earliest evidence of periodontal therapy. Furthermore, prescriptions for alleviating toothache frequently combined magical incantations with medicinal remedies, while several texts also mention instances of dental trauma, highlighting the broad range of oral conditions addressed by these early practitioners.^14^

Within a corpus of diagnostic and prognostic tablets, bruxism was accepted as a symptom of certain diseases. One treatment protocol involved reciting conjurations over a human skull, while another prescribed grinding herbal and mineral substances into a powder, which was then placed in leather pouches to be worn around the neck as prophylactic talismans. A further remedy recommended applying a mixture of amanu*-*salt and juniper directly to the teeth.^15^ Collectively, these practices demonstrate how ancient societies integrated empirical observations with ritualistic elements, forging a multifaceted approach to healthcare that transcended purely physical treatments.

Unfavourable environmental conditions in ancient Mesopotamia have hindered the preservation of skeletal remains, resulting in a limited number of skulls and teeth available for dental analyses.^12^ Among these scarce collections, excavations at the city of Kish uncovered 116 skulls dating to approximately 2000 BC. Studies of the remains have revealed poor dental health: 95% of the specimens showed severe tooth wear, and 42% displayed evidence of periodontal disease, which was often associated with significant calculus deposits; however, carious lesions were rare.^8^^,^^16^ In contrast, a separate study of Bronze and Iron Age sites on the Iranian plateau found a higher prevalence of cervical caries, possibly linked to a diet rich in fermentable carbohydrates such as grains.^8^

General dental anomalies, including tooth crowding and misalignment, were also identified. Enamel hypoplasia was prevalent, indicating periods of nutritional and health stress, likely caused by famine.^17^^,^^18^ However, no evidence of extractions, restorative treatments, or prosthetic dentistry was found at any of the Mesopotamian archaeological sites. Furthermore, surviving texts make no mention of operative treatment.^19^

## Israel

The significance of healthy teeth is highlighted in several references in the Old Testament, where they are regarded as symbols of beauty and strength. In the proverbs of Solomon, bad teeth symbolise weakness. Biblical texts even prescribe punishments for those who cause the loss of teeth: ‘thou shalt give life for life, tooth for tooth, hand for hand…' (Exodus 21: 23–25).^20^

Later writings, particularly in the *Talmud*, offer further insights into dental practices and oral health in ancient Israel. The text describes women adorned with gold crowns and artificial teeth, though no physical examples from this period have been found. While it advises against the unnecessary extraction of teeth, it lacks details on other operative dental procedures. The *Talmud* does, however, mention various remedies for oral health conditions, including vinegar for lacerated gums and treatments for oral pain using sour fruit juice or a mixture of garlic, oil and salt.^21^^,^^22^

The *Talmud* also addresses oral hygiene, recommending a salt poultice for cleaning teeth and gums. To combat bad breath, it suggests adding salt, pepper, or cinnamon to food. The use of toothpicks is mentioned, though it is unclear when this was a widespread practice.^23^^,^^24^ Despite these references, no archaeological evidence of interventive dental treatment has yet been identified for ancient Israel as a whole.

## Etruria and Phoenicia

The study of ancient dentistry has long been enhanced by the dental appliances and bridges unearthed at Etruscan archaeological sites in central Italy. Over the past 150 years, these discoveries have spurred significant academic interest and research, offering a fascinating glimpse into the dental practices of early societies. While the Etruscan civilisation flourished during the first millennium BC, by the 4^th^ century BC, its rich cultural legacy had been gradually assimilated into the expanding influence of ancient Rome (Fig. 1).^25^

Archaeological evidence suggests that Etruscan dental appliances date between the seventh and first centuries BC and were primarily associated with elite women. Approximately 20 such devices have been unearthed, and they seem to have been designed to replace missing anterior teeth, stabilise loose teeth, or serve a cosmetic function. Their usage appears to have declined as Roman influence spread across central Italy.^26^^,^^27^

**Fig. 3:**
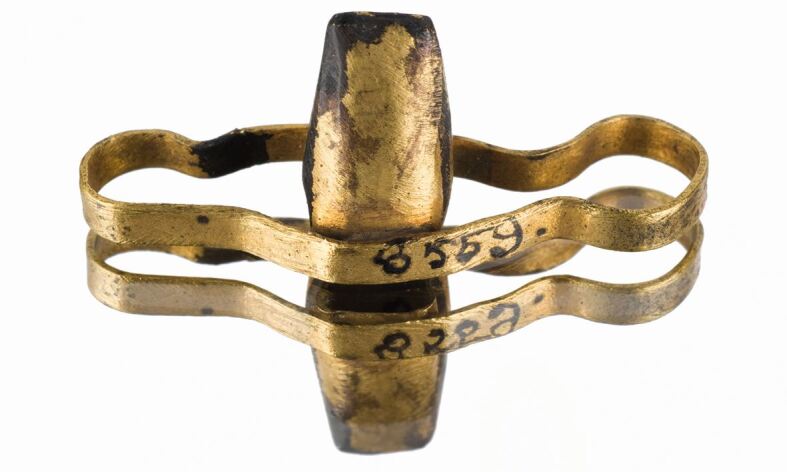
Replica of an Etruscan prosthetic appliance (A622195). ©The Board of Trustees of the Science Museum

**Fig. 4:**
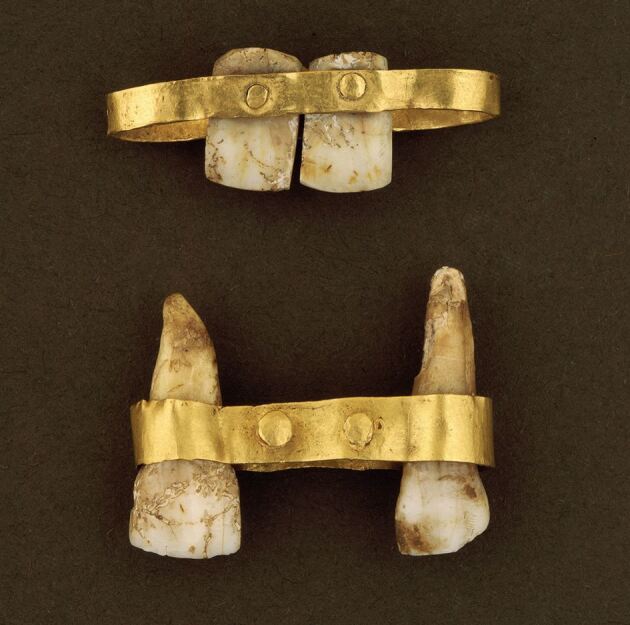
Etruscan bridges. Top: dental prosthesis (M10334). Features two pontics made from trimmed human teeth, possibly the recipient's own, secured to a gold band with rivets. The gold band would have encircled the abutment teeth. Bottom: dental prosthesis (M10335). Originally designed to encircle the four maxillary incisors. The abutment teeth remain, but the two pontics, once held by gold rivets, are missing. Courtesy of National Museums Liverpool, World Museum

The appliances represent true dental bridges, marking the Etruscans as the first civilisation to produce such devices. They used abutment teeth for retainers and constructed pontics to replace up to four missing teeth (Fig. 3, Fig. 4). One common design featured a long oval gold band that closely surrounded a group of teeth, while another employed a series of rings formed from short gold strips cold-welded together, with the lateral loops of the assembly fitting over the abutment teeth. Replacement teeth were fashioned from human or animal teeth with the roots being trimmed and then fixed to the bands by rivets. Unfortunately, most appliances have not survived on their original skulls, making it challenging to determine any details relating to their fit or the condition of the associated teeth.^27^

Nearly all known Etruscan dental appliances replaced maxillary anterior teeth (typically central or lateral incisors); teeth that, notably, were usually among the last to be lost in antiquity. As these devices were primarily worn by elite women and featured elaborate goldwork, some scholars have suggested that teeth may have been deliberately extracted to prominently display the gold work. This theory aligns with the documented practice of dental evulsion or ablation as a cultural custom observed in various societies worldwide.^28^^,^^29^ Consequently, cosmetic concerns likely played a significant role in the adoption of these appliances, as public appearance was highly valued among Etruscan women.

Some scholars have speculated that some of these appliances functioned as rudimentary orthodontic devices, but this interpretation seems unlikely.^30^^,^^31^ The flimsy construction of the Etruscan appliances, such as the ‘Poggio Gaiella appliance' with gold bands only 0.14 mm thick indicates they would not have been able to exert a significant force. Additionally, the absence of accessory wiring or features necessary for tooth movement further undermines this hypothesis. Moreover, none of the appliances were designed for juveniles, who are typically the primary recipients of orthodontic treatment. Instead, ancient texts emphasise their decorative and cosmetic roles.^32^

Two other gold-wire appliances discovered in tombs in Sidon, ancient Phoenicia (modern-day Lebanon and Syria), and dated to approximately 400 BC, have been linked to these Etruscan bridges. Phoenician craftsmen, renowned for their metalwork and jewellery, may have influenced/been influenced by Etruscan artisans.^27^

The number of studies investigating Etruscan dentitions is limited, but surveys have revealed significant patterns of dental wear, comparable to those found in other ancient civilisations. Also, there is evidence of periodontal disease, dental abscesses, premortem tooth loss, and a low caries rate.^33^^,^^34^ Interestingly, these findings indicate that most individuals retained their maxillary incisors at death, providing further evidence in support of the hypothesis that elite Etruscan women may have deliberately undergone dental ablation to facilitate cosmetic replacement with gold dental appliances.

The lack of surviving Etruscan written records has significantly hindered our understanding of their traditions, including potential dental practices. Because Etruscan texts were often written on perishable materials like linen and papyrus, they have not withstood the test of time. As a result, any documentation of dental treatments has been lost, leaving archaeological findings as the primary source of insight into Etruscan oral care.

## Far East

### China

Some of the earliest manifestations of ancient Chinese civilisation emerged in Neolithic and Bronze Age communities along the Yellow and Yangtze River basins. Osteoarchaeological evidence from these regions reveal that teeth were sometimes repurposed as ornamental objects, and there are indications of early, albeit primitive, tooth extraction practices.^35^ The *Nei Ching* (*Classic of Internal Medicine*), conventionally dated to circa 2700 BC and variously attributed either to Huang-Ti, the legendary Yellow Emperor, or to later compilers, stands as one of the oldest known sources of medical and dental knowledge. The *Nei Ching* discusses toothache, dental caries, and periodontal disease.^36^^,^^37^ It also highlights the diagnostic value of tongue examination, considering it as significant as pulse assessment.^38^

Evidence for dental interventions in this early period is fragmentary, but contemporary texts do outline a pharmacopeia of mineral and botanical preparations interwoven with ritual elements. In the *Shiji* (*Records of the Grand Historian*, late 2^nd^ to early 1^st^ century BC), Sima Qian describes a protocol for toothache combining targeted acupuncture with a daily rinse of a bitter root decoction, though the exact herbal formula has been lost to time.^39^ Likewise, *Fifty-Two Prescriptions* – a collection of medical preparations inscribed on silk manuscripts and unearthed from tombs at Mawangdui in the Hunan Province in China (193–168 BC) – includes a remedy comprising finely ground elm bark, *Osmanthus fragrans* and other medicinal substances blended into a lard-based paste. This compound was recommended to be packed directly into carious cavities, functioning as a rudimentary form of a restorative filling.^40^^,^^41^

Few studies have analysed ancient Chinese dental remains. However, examination of cranial remains dated to the Xia Dynasty (c. 2070–1600 BC) reveals a caries prevalence of just 6.9%, substantially lower than the more than 50% prevalence seen in modern populations, and a similarly comparatively low incidence of malocclusion.^42^^,^^43^

No physical evidence of operative interventions has been identified in these, or any subsequent ancient Chinese remains, nor do contemporary texts describe intra-oral surgical procedures. Together, these findings imply that ancient Chinese dental care relied almost entirely on non-invasive approaches, such as external applications and mouthwashes, rather than the restorative or surgical techniques familiar today.

### India

Early references to dental care in India are found in the *Vedas* – sacred Hindu texts written in Sanskrit between approximately 1500 and 500 BC. These texts present a broad spectrum of medical knowledge, including the use of medicinal herbs, hygiene practices and explicit references to oral care.^37^ The *Vedas* likely influenced later medical manuscripts, such as the *Charaka Samhita*, a foundational Sanskrit work on traditional Indian medicine. Along with the *Sushruta Samhita*, these texts form the core of ancient Indian medical knowledge and serve as the basis for contemporary Ayurvedic medicine.^44^ The *Charaka Samhita*, composed between 100 BC and AD 200, provides detailed insights into ancient understandings of the human body and includes therapeutic approaches for a wide range of diseases, while the *Sushruta Samhita* focuses primarily on surgical practices. Both of these works include references to oral and dental conditions.^45^^,^^46^

Periodontal disease is mentioned in these texts, although the ancient understanding of dental plaque and its role in oral diseases appears limited. Recommendations for oral hygiene practices were aimed at not only cleansing the oral cavity but also at prevention. Plants such as tea and lotus leaves were among the substances prescribed for dental care. Modern research has corroborated these practices, demonstrating that lotus leaf extracts^47^ and polyphenols^48^ found in tea exhibit inhibitory effects against pathogens linked to periodontal disease.

Much like traditional Chinese medicine, ancient Indian medical texts emphasise tongue examination as a diagnostic tool. The *Charaka Samhita* dedicates an entire chapter to this practice and advocates tongue cleaning as an essential aspect of personal hygiene. The *Charaka Samhita* also recommends mouthwashes, often infused with sesame oil, aligning with the modern Ayurvedic practice of oil pulling. Additionally, chewing sticks made from medicinal plants such as neem were commonly used for oral hygiene. Today, neem is recognised for its antibacterial, anti-inflammatory and antioxidant properties, which can contribute to improved oral health.^49^

Early Sanskrit literature makes no reference of dental extractions, restorative procedures, or prosthetic treatments, and archaeological evidence similarly fails to corroborate the presence of these practices in ancient India. This absence implies that early Indian dental care prioritised preventive and hygienic measures over surgical and restorative procedures.

A study of approximately 60 individuals from the Indus Valley Civilization (Harappa, c*.* 2500 BC) provides some insight into ancient dental health in the region. The findings revealed significant tooth wear and high frequencies of enamel hypoplasia, consistent with a mixed farming and pastoralist diet. However, moderate levels of calculus, caries and antemortem tooth loss were observed, along with a lower incidence of dental abscesses.^50^

### Japan

The development of dental health practices in early Japan closely mirrored those of other East Asian societies, shaped by the transmission of medical knowledge along the same networks that facilitated the spread of Buddhism and Chinese medicine from China through the Korean peninsula to the Japanese archipelago.^24^^,^^37^ Despite these shared influences, the first Japanese medical text to address oral health – the *Ishinpō* – was not compiled until c. 980 AD by the court physician, Tamba Yasuyori.^51^ Drawing heavily on Chinese sources, it nevertheless offers Japan's earliest detailed account of dental theory and treatment, from herbal mouthrinses to basic tooth extraction techniques. Before its compilation, references to dental care are notably absent in the extant textual record.

Archaeological examinations of Neolithic Jōmon skulls (c. 10500–300 BC) reveal heavy tooth wear caused by a diet rich in coarse grains, nuts and inadvertent sand contamination.^52^ This was a period characterised by hunting, gathering and fishing. Although dental caries were relatively rare in the earliest Jōmon phases, its frequency increased over time, suggesting a gradual dietary shift toward more carbohydrate-rich foods among later communities.^53^

During the Late to Final Jōmon Period (c. 1500–300 BC), selective removal of incisors and canines emerges in skeletal remains from Pacific coastal settlements on Honshu. These extractions, perhaps linked to rites of passage or social status, remain the only clear evidence of intentional dental modification in prehistoric Japan.^54^ So, although dental care in ancient Japan shows clear roots in broader East Asian medical traditions, much remains unknown about oral care practices before the 10^th^ century AD.

## Conclusion

In summary, this paper has highlighted the multifaceted nature of dental care in antiquity, shaped by cultural beliefs, dietary habits and early technological innovations. From preventive measures, such as chew sticks and mouthwashes, to rudimentary restorations, these interventions laid foundations for the evolution of modern dentistry. Nevertheless, a truly comprehensive picture will only emerge through further interdisciplinary collaboration, integrating close readings of ancient texts, systematic archaeological investigation, and bio-anthropological analysis.

Part two of this study will extend this inquiry to the dental traditions of ancient Egypt and the Graeco-Roman world, deepening our comparative understanding of how early civilisations addressed oral health and set the stage for later advances.
